# Sustaining health systems in sub-Saharan Africa: public–private partnerships in a new era of reduced donor funding

**DOI:** 10.1093/heapol/czag008

**Published:** 2026-02-05

**Authors:** Rowan Haffner, Faraan O Rahim, Lara Kendall, Sarah Ali, Rohith Karthik, Ketan Tamirisa, Mahmood Abdelkader, Abebe Bekele

**Affiliations:** Department of Neuroscience, Duke University, 417 Chapel Drive, Durham, NC 27708, United States; Harvard Medical School, 25 Shattuck Street, Boston, MA 02115, United States; Department of Neuroscience, Duke University, 417 Chapel Drive, Durham, NC 27708, United States; Department of Biology, University of Pittsburgh, 4200 Fifth Avenue, Pittsburgh, PA 15260, United States; Department of Nutritional Sciences, Cornell University, 410 Thurston Avenue, Ithaca, NY 14850, United States; Department of Biology, Washington University in St. Louis, 660 South Euclid Avenue, St. Louis, MO 63110, United States; Department of Biology, University of Pittsburgh, 4200 Fifth Avenue, Pittsburgh, PA 15260, United States; Department of Surgery, University of Global Health Equity, Butaro, Burera District 350, Rwanda

**Keywords:** health system strengthening, global health, Africa

## Abstract

Recent reductions in US global health funding have disrupted essential programs in sub-Saharan Africa, highlighting the region’s vulnerability to external financing shocks. The suspension of the United States Agency for International Development initiatives has affected disease control, maternal care, and health system operations across 47 countries, raising urgent questions about how to sustain progress without reliable donor support. This commentary examines the potential of public–private partnerships (PPPs)—structured collaborations in which governments and private actors share financing, risk, and managerial responsibility—to strengthen domestic capacity. Drawing on examples from Senegal, Nigeria, and Kenya, we explore how service-, concession-, financing-, and technology-focused PPPs can mobilize additional resources, expand access, and improve service delivery. We also address key challenges, including governance risks, fiscal constraints, and shifting global power dynamics. While not a substitute for aid, well-designed PPPs aligned with national priorities can support more resilient, equitable, and self-reliant health systems in sub-Saharan Africa.

Key messages

- Shrinking donor funding in sub-Saharan Africa demands investment in resilient infrastructure and local capacity to build sustainable health systems.
- Well-designed public–private partnerships (PPPs) can mobilize resources, expand services, and drive innovation in alignment with national priorities.
- Case studies from countries like Nigeria illustrate how PPPs can strengthen health equity and resilience, but also expose risks without strong governance.
- Effective PPPs require clear accountability, oversight, and community engagement to prevent inequities and ensure lasting improvements.

## Introduction

Recent shifts in US foreign policy have sharply reduced global health support to sub-Saharan Africa (SSA). These actions include the US withdrawal from the World Health Organization and suspension of foreign aid for review, which disrupted long-standing partnerships (The White House 2025). Although a temporary waiver protected the President’s Emergency Plan for AIDS Relief, the United States Agency for International Development, a central actor in disease control and health systems support across 47 SSA countries, was effectively shut down, terminating 5200 of its 6200 programs and placing 4700 employees on administrative leave (Kates 2025). The cuts also halted programs related to malnutrition, food assistance, and malaria control in countries such as Congo, Ethiopia, and Senegal (Knickmeyer et al. 2025).

Foreign aid has historically been integral to health gains in SSA. In 2022, the USA provided 5.84 billion dollars for HIV and AIDS, malaria, maternal and child health, and disease prevention (Husted et al. 2023). The President’s Emergency Plan for AIDS Relief has contributed to >26 million lives saved, supported millions of HIV-free births, and helped expand specialist physician capacity in Rwanda (Able-Thomas et al. 2024). With this foundation now uncertain, an urgent question arises: how can health system progress be sustained without reliable external funding?

One proposed mechanism is the public–private partnership (PPP) model. PPPs are structured collaborations in which governments and private actors share financing, risk, and managerial responsibility (Grimsey and Lewis 2004). This commentary evaluates whether PPPs can serve as a viable pathway from aid dependence to self-reliance and focuses specifically on PPPs that influence health-service delivery, financing, and infrastructure.

## From aid dependence to self-reliance: why PPPs matter

Sustaining health gains in SSA requires systems that can function independently of external aid. Long-term resilience depends on reliable domestic financing, strong infrastructure, and a well-trained workforce, along with supply chains and service networks that can continue operating during political or economic shocks (Baynes et al. 2022). As countries face demographic transitions, infectious threats, and rapid technological change, health systems must integrate new tools, support local research, and respond flexibly to emerging needs (Coiera and Hovenga 2007). Strong systems also contribute to social and economic stability by reducing preventable disease burdens and protecting households from catastrophic health expenditures (Kruk et al. 2015, Thomas et al. 2020). The 2014 Ebola outbreak provided clear evidence of this, as fragile systems amplified indirect mortality when routine services collapsed (Heymann et al. 2015).

The contraction of donor support highlights the urgency of identifying mechanisms that strengthen domestic capacity. PPPs offer one such pathway. PPPs can mobilize resources in contexts where health sectors in SSA face large financing gaps, such as the 25 to 30 billion dollars needed for hospital and distribution networks over the coming decade (International Finance Corporation 2007). They also bring managerial expertise, technological innovation, and operational efficiencies which improve service delivery and enable governments to maintain essential functions during instability (Alonazi 2017, Strasser et al. 2021). When aligned with national priorities, PPPs support infrastructure investment, expand access to essential services, and contribute to workforce development.

## Illustrative PPP models in practice

Experiences across sub-Saharan Africa show how service-, concession-, financing-, and technology-focused PPPs can strengthen health systems when designed and governed effectively. In Senegal, a long-running service PPP for urban water delivery expanded household connections and increased coverage for low-income communities without reliance on US donor leadership, demonstrating how structured contracts can improve essential infrastructure (Marin et al. 2025). In Nigeria, the Federal Capital Territory Administration partnered with Nisa Premier Hospital through a concession agreement to rehabilitate and manage Garki Hospital, leading to major modernization, expanded specialist training programs, and investments in advanced equipment. By 2017, the hospital had completed Nigeria’s first successful kidney transplant and strengthened its postgraduate training capacity (Anthony 2017). Nigeria’s Private Sector Health Alliance (PSHAN) illustrates a domestically led, financing-oriented PPP model: PSHAN mobilizes corporate and philanthropic contributions to support national health priorities, alongside performance-based public financing reforms such as the World Bank’s Saving One Million Lives Program-for-Results (World Bank 2021). Together, these features show how domestically led PPPs like PSHAN can complement public financing reforms and help maintain progress as external donor support declines.

Technology partnerships also expanded access during Kenya’s COVID-19 response, where collaboration between PharmAccess and local agencies increased PCR testing capacity and introduced real-time digital tracking systems, showing how PPPs can rapidly mobilize technical expertise during emergencies (van Duijn et al. 2023). Comparable PPP initiatives have demonstrated similar gains in technology adoption (Ebalue et al. 2024). Together, these models demonstrate how PPPs improve service delivery, including mobilizing non-state financing, strengthening management capacity, and accelerating technology adoption.

## The additionality debate

A central question is whether PPPs generate new resources or simply repackage future public spending. While many PPPs involve government repayment of private investment, several forms of additionality have been documented. Philanthropic or corporate contributions can supplement state budgets, and efficiency gains or risk transfers can reduce costs and create fiscal space, showing how PPPs can generate real additionality when they bring new actors or reduce inefficiencies (Quiggin 2004, Sinanovic and Kumaranayake 2006)

## Opportunities, challenges, and future directions

PPPs offer several opportunities to strengthen health systems, particularly when they expand resources, introduce new technologies, and support local capacity. Technology-focused collaborations provide one example. Models such as the Health Equity Consortium show how shared data systems and mapping tools can help local providers identify gaps in care and reach underserved communities, when supported by strong local leadership (Arnaout et al. 2023). New financing approaches also offer promise. Outcome-based and risk-sharing arrangements can align incentives and reduce financial pressures governments face under fixed-price contracts, which limit flexibility and can shift risk onto the public sector (Liu et al. 2024).

At the same time, PPPs bring challenges that require careful governance. Although PPPs offer important opportunities, their benefits are not automatic. If agreements are poorly designed, they can draw funding away from essential services or create long-term financial burdens. The partnership between the Ministry of Health in Lesotho and the Queen Mamohato Memorial Hospital illustrates this risk, as improved service quality came alongside high costs that strained the health budget and left fewer resources for rural primary care (Hellowell 2019). Clear contracting, regular audits, and public reporting are essential for accountability and public trust (Reich 2018). Looking ahead, the broader global financing landscape must also be considered. As traditional donors withdraw, gaps in funding are increasingly filled through arrangements with external actors, including China’s concessional loans and infrastructure investments (Chinese Infrastructure Lending and Africa’s Global Value Chain Participation 2024). Strong negotiation capacity and government leadership are critical to ensure PPPs strengthen sovereignty, equity, and long-term resilience rather than create new dependencies.

## Conclusion

As foreign aid declines, countries in SSA must identify sustainable ways to protect recent health gains and strengthen domestic systems. PPPs are not a complete substitute for aid, but they can play a meaningful role when aligned with national priorities, carefully governed and designed to promote equity and long-term capacity. Their potential lies in supporting infrastructure, expanding access, and fostering innovation while maintaining strong public oversight. With safeguards in place, PPPs can play a meaningful role in building resilient and increasingly self-reliant health systems in SSA.

## Data Availability

References used in the writing of the manuscript are available from the authors.

## Reflexivity statement

The authors represent a diverse and multidisciplinary team across eight institutions in North America and East Africa. The group includes both male and female contributors spanning early-career researchers, mid-level professionals, and senior faculty members. Disciplines represented include medicine, public health policy, and health systems research. While the majority of the authors are based at institutions in the United States, the corresponding author is based in Rwanda and brings extensive leadership experience in health-system strengthening across Sub-Saharan Africa. This diversity of gender, geography, and seniority has enriched the analysis and interpretation of public–private pmodels within varied African health contexts.
